# Imponderable Agents

**Published:** 1833

**Authors:** 


### Art. IX. Imponderable Agents.

*Elements of Electricity and Magnetism, and Electro-Magnetism; embracing the late discoveries and improvements, digested into the form of a Treatise.* By John Farrar, Prof. Harvard University. Cambridge, 1826.

Some departments of human knowledge are confined within narrow bounds, and have, comparatively, but few relations with the rest. They may be studied without the aid of many others; and, like the Caspian Sea, can be explored to their utmost limits, without the preparation or the events of a lengthened voyage. Others connect themselves with almost every department, and their extended limits, like those of the ocean, run into the most distant recesses of science. Of this kind is the study of man, in his physiological and pathological states of existence. Hence, we find, that to be profound physiologists and physicians, it is indispensable that we should direct our attention upon almost every branch of natural, and indeed moral science. Man, in reference to the agents and influences which act upon him, may be said to occupy a focal spot in the creation;—to be, as it were, in the very centre of gravity—having every surrounding object in action upon him, as all bodies press towards the centre of the earth. By the instrumentality of these various agents, exerting themselves according to fixed laws imposed on them by the Creator, *he lives, moves, and has his being;* but while, in their regulated action, they sustain him from birth to death, and administer to his enjoyments, their excessive, defective and untimely operation, are the chief, if not the sole causes, of the various bodily calamities, which so often cripple his frame, pervert his complicated functions, and terminate prematurely his existence. All his knowledge of the sinister effects of these agents upon his system, is the result of experience. *A priori*, he could know little, even of their ordinary influences; and still less could he comprehend, without long-continued observation and experiment, in what way they are deeply and essentially concerned in the production of the phenomena of life. And still, in the progress of discovery, it has been revealed to him, that several of these agents *are* the immediate causes of all his vital functions. From the beginning it must have been known, that in addition to food and drink, two subtle and invisible agents which surround him, are indispensable to the continuance of life. These are caloric and atmospheric air, or its oxygen. Within our own times, a number of facts have been developed, which seem to indicate, that electricity, galvanism, magnetism and light, (whether considered as distinct substances, or as modifications of one,) are in some unknown—perhaps incomprehensible—manner, concerned in producing the phenomena of life. We must not be understood to intimate a belief, that oxygen, or caloric, or electricity, is the *primum mobile*, of animated existence; for we know of no effects, produced by any or all of those agents, which would justify us in such an assertion. The phenomena of intellection, volition, sensation, and even organization, appear clearly to indicate some higher, though hidden principle—hidden except by its effects;—and that oxygen, caloric and electricity, are but operatives in its service; operatives, however, without which it could make no manifestation. It being ascertained, that these gaseous or imponderable substances, play an important part in the performance of our functions; we must admit, *in extenso*, the propriety of studying the laws which govern their actions, among the various objects of nature; and, especially, their influence on man. Thus every physiologist and physician, should, to a certain extent, be a chemist and a natural philosopher. Not endowed with the knowledge of a manufacturer of acids and air-pumps, but familiarly acquainted with the *modus agendi* of oxygen, caloric, light, galvanism, electricity and magnetism, on the living system. To this end, chemistry and the rudiments of natural philosophy, have been made essential branches of study, in all our medical schools; but we are sorry to say, that notwithstanding this regulation, they are too often regarded as unimportant; and that a majority of the faculty (including ourselves) enter, sojourn and leave, the ranks of the profession, with far less knowledge of these interesting subjects, than of the qualities and capacities of the persons, whose names are recorded in our *ledgers*. This glaring defect, like most professional imperfections, is attributable to our teachers. Very few of them have, at any time, had more than a superficial knowledge of these matters, and the stream cannot rise higher than its fountain. It is to raise the waters of the latter, that we indite these paragraphs, having placed at the head of this article, the name of a standard work which belongs to the curious and difficult department, which is so generally made a *pons asinorum*, by our students. We are far from presuming to think, that any exhortation, or any philippic of ours, can oxiginate that which has no affinity for oxygen, excite that which cannot be electrified, or infuse polarity into what cannot be magnetized; but there are grades of intellectual hebetude, and although we may not rouse the sluggard, whom no summer can thaw out of his *hybernaculum*, we may hope to excite some galvanic contractions, and impart some impulse, to the idler, provided he have but industry and enterprise enough, to subscribe for our journal, and read what is here addressed to him. That students and physicians, in a single year, waste more time than would be necessary to acquire a knowledge of all the facts, individual and general, which have been developed by the philosophers who have cultivated these abstruse departments of science, we hold to be a more incontrovertible proposition, than most which belong to the profession; and we would respectfully put to them the question,whether this negligence is compatible, either with their own dignity, or the safety of their patients. Should any one doubt, whether there is "room for improvement" among us, let him reflect on the indications that are afforded by such inquiries and suggestions as these, which may be given as specimens:—"That oxygen and caloric are commutable, and that the former, being taken into the blood through the lungs, is transformed into the latter, and constitutes animal heat;—that there are two kinds of electricity, the resinous and the negative;—that cold is the antagonizing power to heat, and that there are two kinds of caloric, one of which is positive, the other negative;—that magnetism is probably a modification of cold;—that the difference between the electrical machine and the galvanic pile, may, perhaps, be this, that one generates vitreous, and the other resinous electricity; finally, that shooting-stars are owing to the combustion of carbonic acid gas."

Mistakes of this kind, are not so much errors of fact, as of principle; and indicate an ignorance of several branches of physical science, which is truly discreditable to the profession. We have said that the fault is with our teachers, but should have added—not our medical teachers only. It is quite impossible to study the laws of light, caloric, electricity and magnetism, without some previous acquaintance with mathematical science, an attainment within the reach of very few who are put to the study of medicine. It is notorious, that the greater number, indeed a large majority of our medical pupils, become such, without the least knowledge of geometry, algebra or mechanics, from never having been placed in academies where they are taught; and even those who pass through a college course in most of our institutions, some how or other, are protruded from them, with the exact sciences spread over the *surfaces* of their minds, as electricity is diffused *upon* bodies; and they lose it about as soon. We seriously doubt, whether a tythe of the profession, junior and senior, in the United States, are able, for want of preparatory learning, to read the works, a specimen of which is placed at the head of this article.

That treatise, bearing on its face the name of Professor Farrar, of Harvard University, is a translation from the *precis Elementaire de Physique* of M. Biot, a distinguished French mathematician and natural philosopher. Were we competent, we should not undertake an analysis of this able production, for our leading object, is merely to excite the younger members of the profession, to a profounder study of the subjects which it embraces. But we may be permitted to make a few extracts, as specimens of the work. It has been commonly said, that electricity accumulates on the surface of non-conductors, but penetrates conductors in all directions. M. Biot thought this opinion worthy of being investigated, and has accordingly tested it by a series of experiments. He was induced, *a priori*, to believe that the fluid occupies the surface only, of conductors as well as non-conductors. These are his words:

"Now, from what we have already learned upon this subject, it would seem very probable that the electricity is confined entirely to the surface of conducting bodies, and that their interior particles have no effect in retaining it; otherwise, it is not easy to perceive how the mere circumstance of equality of surface in the case of two bodies in contact, should produce between them an equal division of electricity, whatever be the substance of the bodies themselves; or how this equality should take place, when one of the bodies is solid and compact, and the other hollow and presenting scarcely any thing but a simple surface; whereas all these things become simple and intelligible, on the supposition that the electricity, in a state of equilibrium, is diffused only over the surfaces of bodies, without penetrating into the interior."

Passing over the experiments by which this fact was demonstrated, we shall transcribe his conclusion:

"We may rest assured, therefore, that the electric principle, whatever it may be, resides on the surface of conducting bodies, and not in their interior. We know, moreover, by further experiments, that the air retains it upon the surface, and is the only obstacle which prevents its escape from the body. Hence, combining these two facts, it will be seen, that the electric principle always distributes itself over conducting bodies in a very thin stratum, whose exterior surface being contiguous to the air, and confined by the pressure of this fluid, is the same as that of the electrified body, while the surface, almost coincident with the other, since the stratum is very thin, must be determined according to other laws, to be deduced from observation."

The electric fluid, then, is found only on the surfaces of bodies, and this is as true of conductors as non-conductors.

The difference between the two is simply this, that it moves, on the former, with the utmost freedom, and may all pass off through a single point, if that point be connected with the earth; but it travels with great difficulty, or not at all, on the surface of non-conductors.

The following extract will present the conclusion of our author on another interesting point in the science of electricity:

"We hence learn, that the principles of the two electricities exist naturally in all conducting bodies, in a state of combination by which their effect is neutralized; this we shall in future call *the natural state of bodies.* We now perceive that friction, which seemed to be a means of creating them, serves only to disengage them from their combination, and to render one of them sensible by absorbing the other; and this is the reason, undoubtedly, why we constantly observe that the rubbing body and the body rubbed, exhibit contrary electricities. Finally, since the simple influence of an electrified body, presented at a distance, forces these two electricities to separate, and to arrange themselves so that those of a different nature are the nearest to each other, and those of the same nature the most remote, in enunciating this fact, we are compelled to admit, *that opposite electricities attract, and similar electricities repel each other*, according to laws which we shall be able to determine by experiment."

From electricity, Biot proceeds to galvanism, then to magnetism, and concludes with the deeply interesting phenomena of electro-magnetism. We commend the whole to such of our readers as would prepare themselves for studying the influence of these powerful agents on the animal economy.

Professor Farrar has added to the volume a number of notes, one of which is an extract from a memoir of M. Gay-Lussac, on the construction of lightning rods; a subject with which every physician should be acquainted, as he is liable to be consulted by those who presume that he is able to give correct advice. We shall make a condensed abridgement of these instructions.

1. The lightning rod should be a cone or pyramid, divided at the top into several points, and the higher it is elevated into the atmosphere, the greater, *ceteris paribus*, will be its efficacy. It will protect a circular area, the diameter of which is four times the length of the rod. It should rise from 15 to 20 feet above the top of the building.

2. It should be so thick, that the wind acting upon this upper part, cannot bend it, and present sufficient surface to transmit the discharge from the cloud, without being fused or permitting any of the electricity to pass into the building. A rod from half an inch to three-quarters in breadth, will be of sufficient size; though the lower part should be of greater dimensions.

3. It should always terminate in *moist* ground, as dry earth is a non-conductor. After sinking about three feet into the earth, it should extend off horizontally, 12 or 15, and, if practicable, terminate in a drain or gutter; if not, it should be sunk several feet deeper into the ground, and, throughout its whole extent, be surrounded with charcoal, which will not only protect it from rust, but carry off the electricity. If its extremity be made to terminate in several prongs so much the better.

4. If the rod be composed of several pieces, they should be so joined together, as to keep them in close and permanent contact.

5. The points of the rod should be composed of copper, plated with silver, as iron is liable to become oxidated.

When a discharge of electricity takes place from a cloud to the earth, it is because the two are in opposite states, as to that fluid—one vitreous and the other resinous, or positive and negative. If a house happen to stand on a portion of the earth thus charged with an electricity different from that of the cloud, a discharge from the latter to the former, may fall on the building. If it have no conductor, it may be injured and its inhabitants destroyed: if it have a rod, formed and fixed according to the instructions given above, it may be, unhesitatingly, said, the edifice is safe. The rod, does not bring down the lightning from the cloud, but only seizes upon and conducts it to the earth, when in descending, it happens to pass near the building. We can, at this moment, recollect three discharges, in this city, which reached the ground through very humble objects, quite contiguous, to others of the same kind, which were much loftier; showing that, it is the condition of the earth, and not the attraction of the objects on its surface, that causes the discharge.

